# The effect of miR-205a with RUNX2 towards proliferation and differentiation of chicken chondrocytes in thiram-induced tibial dyschondroplasia

**DOI:** 10.1016/j.psj.2024.104535

**Published:** 2024-11-08

**Authors:** Yuxin Zhou, Yuxiang Lu, Hengyong Xu, Xuyang Ji, Qingqing Deng, Xi Wang, Yao Zhang, Qiuhang Li, Yusheng Lu, Alma Rustempasic, Yiping Liu, Yan Wang

**Affiliations:** a State Key Laboratory of Swine and Poultry Breeding Industry, College of Animal Science and Technology, Sichuan Agricultural University, Chengdu 611130, PR China; b Farm Animal Genetic Resources Exploration and Innovation Key Laboratory of Sichuan Province, Sichuan Agricultural University, Chengdu Campus, Chengdu 611130, PR China; c Key Laboratory of Livestock and Poultry Multi-omics, Ministry of Agriculture and Rural Affairs, College of Animal Science and Technology, Sichuan Agricultural University, Chengdu 611130, PR China; d Faculty of Agriculture and Food Science, University in Sarajevo, Zmaja od Bosne 8, 71000 Sarajevo, Bosnia and Herzegovina

**Keywords:** miR-205a, *RUNX2*, Chicken, Chondrocytes, Tibial dyschondroplasia

## Abstract

Tibial dyschondroplasia (**TD**) is a kind of metabolic bone disease in fast-growing broilers, which seriously restricts the development of poultry industry. Our previous studies have revealed a significant upregulation of miR-205a in TD cartilage tissue, suggesting its potential role as a regulatory factor in the pathogenesis of TD. However, the precise function implications and underlying regulatory mechanism remain elusive. Therefore, this study aims to elucidate the biological functions and regulatory mechanisms of miR-205a in the progression of TD by employing mehtodologies such as qRT-PCR, CCK-8 assay, EdU assays, and flow cytometry. The findings demonstrated that the transfection of miR-205a overexpression plasmid reduced chondrocytes growth and development in TD while enhancing apoptosis; conversely, blocking miR-205a had opposite effects. *RUNX2* was identified as a target gene of miR-205a through biosynthesis and dual luciferase assays, and its overexpression helps chondrocytes in TD grow and develop. However, when both miR-205a and *RUNX2* were overexpressed, the regulatory effect of *RUNX2* was significantly suppressed. In conclusion, miR-205a plays a role in slowing the growth and development of chondrocytes in TD by targeting and reducing *RUNX2* expression, which helps to initiate and progress TD.

## Introduction

It is well known that poultry bones generally have the function of protecting organs, supporting weight, promoting movement and maintaining mineral balance (Bar, 2009; Sharma et al., 2023). Recently, commercial broilers under intensive conditions have a higher growth rate and feed conversion rate, which can meet consumer demand for chicken. However, the rapid growth causes the skeletal system to have difficulty adapting to the weight gain, eventually leading to lameness and bone disease in broilers, which not only causes significant losses to the breeding industry, but also adversely affects animal welfare (Baxter et al., 2021; Cai et al., 2023).

The pathologically features of TD in broilers are typically characterized by the presence of a white, transparent, non-mineralized cartilage plugs within the proximal growth plate (Pelicia et al., 2012; Tian et al., 2013; Huang et al., 2018). According to statistics, 30% of poultry bone diseases are caused by TD, and the incidence in China is also between 5% and 10%, especially in Jiangsu, Shandong, Liaoning and Hebei (Mehmood et al., 2018; Zhang et al., 2018). Recently, researchers have sought to investigate the pathogenesis of TD through the examination of related genes (Yao et al., 2020), potential pathways, and intestinal microbes, with the aim of enhancing leg health in poultry and reducing the incidence of TD (Huang et al., 2022; Mo et al., 2023; Xu et al., 2023). For instance, Jahejo et al. (2019) identified that angiogenesis-related genes and MAPK signaling pathway genes, including *RAC2, MAP3K1, PRKCB, FLNB*, and *IL1R1,* are downregulated in thiram-induced TD, suggesting their significant role in regulating the onset of TD. Additionally, Yao et al. (2018) observed that total flavonoids derived from rhizoma drynariae could upregulate BMP-2 and RUNX2 expression levels in cases of TD. This modulation appears to alleviate the condition while improving production performance among affected chickens. However, these studies have primarily concentrated on a single aspect and do not comprehensively elucidate the molecular mechanisms underlying the occurrence of TD. Consequently, the prevention and treatment of TD continue to pose significant challenges in broiler production.

MicroRNAs (**miRNAs**) are small, naturally occurring non-coding RNAs that are about 23 nucleotides long and can attach to the 3′ untranslated region (**3′ UTR**) of mRNAs. This attachment can either cause mRNA to break down or prevent it from being translated (Rebucci et al., 2015; Ran et al., 2023). Importantly, miRNAs play critical regulatory roles in the onset and progression of bone diseases by promoting cartilage formation and modulating chondrocyte differentiation (Melnik et al., 2020; Tang et al., 2008a). For instance, Cao et al. (2020) demonstrated that miR-296-5p is lower in human chondrocytes treated with IL-1β and can help prevent chondrocyte apoptosis and cartilage breakdown by directly targeting the TGF-β1/CTGF/p38MAPK signaling pathway. Similarly, Oka et al. (2020) found that miR-21 positively regulates osteoblast differentiation and bone mineralization by promoting the expression of *ALP, MEF2C, RUNX2*, and *OSX*. Many studies have shown that miR-205 is not just related to the development of different types of cancer but is also connected to bone diseases (Zhao et al., 2021; Gan et al., 2023). For example, the level of miRNA-205 in bone metastatic prostate cancer is lower than that in non-bone metastatic prostate cancer, demonstrating significant differentiation capability and indicating its crucial role in the tumorigenesis and bone metastasis of prostate cancer (Sun et al., 2020). Furthermore, recent studies have demonstrated that inhibition of miR-205 enhances bone formation in bone marrow mesenchymal stem cells while negatively regulating osteogenic differentiation (Hu et al., 2015). All these findings have demonstrated the important regulatory role of miR-205 in bone formation and disease, but its specific role and regulatory mechanism in the pathogenesis of TD in chickens remain unclear.

Runt-related transcription factor 2 (**RUNX2**), one of three members of the RUNX family, is a crucial transcription factor for bone development. It regulates the expression of various extracellular matrix protein genes during the differentiation of chondrocytes and osteoblasts (Komori, 2010; 2022). Previous research has shown that RUNX2 is regulated by a variety of miRNAs, which play a significant role in bone development and formation (Narayanan et al., 2019). For instance, Meng and his colleagues (2018) found that miR-193b-3p regulates chondrogenesis by directly targeting *RUNX2*. Similarly, miR-505 has also been shown to partially regulate osteoblast differentiation dysfunction by binding to the *RUNX2* gene (Li et al., 2020). Most importantly, miR-205 can directly inhibit the expression of its target gene RUNX2. Therefore, miR-205/*RUNX2* may represent a novel therapeutic target for elderly women with type 2 diabetes mellitus and osteoporosis (Zhang et al., 2020). However, it remains uncertain whether miR-205 can regulate TD in poultry by targeting *RUNX2*.

Therefore, the goal of this study was to look into how miR-205a and *RUNX2* affect the growth, apoptosis, and differentiation of TD chondrocytes, as well as to explore how they interact with each other. These results are expected to provide valuable insights for the prevention and control of TD, and provide targets and ideas for the breeding of TD-resistant chickens.

## Materials and methods

All animal experiments and procedures conducted in this study were approved by the Animal Care and Use Committee of Sichuan Agricultural University, Sichuan, China, under Permit No. 2014-18.

### Animal and experimental design

Fifty healthy one-day-old Tianfu broilers with comparable body weights were sourced from the poultry breeding farm of Sichuan Agricultural University (Ya'an, China), and the experiments were conducted over a duration of 10 days. All broilers were raised in identical environments and had unrestricted access to food and water. At 5 days of age, the chickens were randomly split into two groups (n=25): (1) the control group, which was given a standard diet with unlimited water; and (2) the TD group, which received a standard diet with added with 50 mg/kg of thiram (purity ≥ 98%, #C10036460, Macklin Biochemical Co., Ltd., Shanghai, China). The chickens in the TD group were monitored daily for lameness symptoms, which became apparent at 10 days of age. At this point, chondrocyte collection and culture were performed.

### Cell isolation and culture

Chicken primary tibial chondrocytes were isolated and cultured according to our pre-optimized method (Ye et al., 2019). In briefly, the isolated proximal tibial white cartilage tissue of the right and left sides of the chicken was temporarily placed in a sterile petri dish containing 1% penicillin-streptomycin (Solarbio) phosphate buffered (**PBS**, Solarbio, Beijing, China). They were then cut into square millimeter particles and digested with 0.25% trypsin (Gibco, Grand Island, NY) and 0.15% type II collagenase (Gibco) for 20 min and 2 h, respectively, to obtain chondrocytes. Subsequently, the chondrocytes were added at a density of 2 × 10^5^ cells per milliliter into a complete medium made up of 10% fetal bovine serum (FBS, Gibco, Grand Island, NY), 1% penicillin-streptomycin (Gibco), and 89% DMEM/F-12 (Gibco). They were kept in a cell incubator at 37°C with 5% CO_2_.

### Immunofluorescence staining

To identify the isolated cells as chondrocytes, we performed immunofluorescence experiments using chondrocyte specific antibodies according to our previously published method (Xu et al., 2024). Specifically, when the density of chondrocytes in the 6-well plate reached 60-70%, the cells were washed with sterile PBS 3 times for 5 min each. Next, the cells were fixed with 4% paraformaldehyde for 30 min and washed 3 times for 3 minutes each. After fixation, the cells were treated with Triton X-100 (Gibco) at a concentration of 0.1% for 20 min to make them permeable. Finally, after washing three times with PBS, the cells were blocked at 37°C for 2 h using 10% goat serum (Solarbio). After washing off the excess goat serum, 500 μL of type II collagen antibody (1:100 dilution; Abcam, USA) was added and left to incubate overnight at 4°C. After three washes with PBS, the cells were then incubated with 200 μL of PBS containing IgG antibody (1:250 dilution; Abcam, USA) at 37°C away from light for 2 h. The cells were stained with 4′,6-diamidino-2-phenylindole (**DAPI**, Gibco) in a dark environment for 5 min. After sealing each cell with drops of glycerol, we looked at the samples under a fluorescence microscope (Eclipse E400, Nikon, Japan) at 400 × magnification, examining three random fields of view for each sample.

### Target Gene Prediction of miR-205a

miR-205a is a highly expressed miRNA found in the TD group in our previous sequencing, indicating that it may be an important miRNA affecting the occurrence of TD (Lu et al., 2022). Therefore, we use online RNAhybrid (http://bibiserv2.cebitec.uni-bielefeld.de/rnahybrid/) and miRDB (http://mirdb.org/miRDB/) tools to make bioinformatic predictions of their potential target genes.

### Dual-luciferase reporter assay

To check if *RUNX2* is a target gene of miR-205a, we seeded DF-1 cells into 24-well plates until they reached about 60%-70% density. We co-transfected these cells with either miR-205a mimics or corresponding negative control (**NC**) along with specific luciferase reporter plasmids that had wild-type (**WT**) or mutant (**Mut**) target sequences made by Sangon Biotechnology Co., Ltd. (Shanghai, China). After a 48 h incubation, the relative luciferase activity of the cells was assessed in accordance with the instructions provided by the Dual Luciferase Reporter Assay System kit (USA).

### Cell transfection

When cell confluence reached 60%-70%, the cells were transfected with miR-205a mimics, miR-205a inhibitors, negative control (**NC**) mimics, inhibitor NC, *RUNX2* overexpression vector (**pcDNA3.1-*RUNX2***) and *RUNX2* small interfering RNA (**si-*RUNX2***), following the product instructions for Hieff Trans® Universal Transfection Reagent (Yeasen, Shanghai, China). After 24 h transfection, chicken TD chondrocytes were used for follow-up tests. The aforementioned substances and their negative controls were synthesized by Sangon Biotechnology Co., Ltd (Shanghai, China), and the sequences information is presented in Table 1.

**Table 1:** Oligonucleotide sequences used in this study.

Name		Sequences (5’-3’)
gga-miR-205a mimics	Sense	UGGAAUGUAAAGAAGUAUGUA
	Antisense	CAUACUUCUUUACAUUCCAUU
mimics NC	Sense	UUCUCCGAACGUGUCACGUTT
	Antisense	ACGUGACACGUUCGGAGAATT
gga-miR-205a inhibitor	Sense	UACAUACUUCUUUACAUUCCA
inhibitor NC	Sense	CAGUACUUUUGUGUAGUACAA
si-RUNX2	Sense	GAAGCUUGAUGACUCUAAATT
	Antisense	UUUAGAGUCAUCAAGCUUCTT
siRNA NC	Sence	UUCUCCGAACGUGUCACGUTT
	Antisense	ACGUGACACGUUCGGAGAATT

### RNA Extraction and real-time quantitative PCR (qRT-PCR)

We used Trizol reagent (Takara, Dalian, China) to extract total RNA from chicken chondrocytes and cartilage tissues according to the manufacturer's instructions, and then we checked the concentration and purity of the RNA with a Nanodrop 2000 (Thermo Fisher, Waltham, MA). Once the RNA passed the concentration and purity tests, we used the PrimeScript RT Master Mix (TaKaRa) reverse transcription kit to turn it into cDNA. We carried out qRT-PCR using SYBR Green Master Mix (Vazyme, Nanjing, China) on a Bio-Rad CFX96 real-time PCR system (Bio-Rad Laboratories, Inc., Shanghai, China). The total reaction volume was 10 µL, which included 5 µL of SYBR Green Master Mix, 0.5 µL each of forward and reverse primers, 1.5 µL of cDNA, and 2.5 µL of RNase-free water. The qRT-PCR amplification conditions were as follows: 98°C 40 s, 98°C 15 s, annealing temperature 20 s, 72°C 35 s, 40 cycles, 72°C 4 min, 4°C preservations for detecting. U6 and *GAPDH* served as internal control for miRNA and other genes, respectively. Each experiment was conducted in triplicate, and the 2^-ΔΔCt^ method was employed to analyze the relative expression levels of qRT-PCR data (Livak and Schmittgen, 2001). All primers were synthesized by Sangon Biotech (Shanghai, China), with their sequence information provided in Table 2.

**Table 2:** Primers used for RT-qPCR.

Gene	Sequences (5’-3’)	Tm (°C)	Product length (pb)
GADPH	F: GGTGGCCATCAATGATCCCT	59	105
	R: CCGTTCTCAGCCTTGACAGT		
CyclinD1	F: TGTCGTTCGAACCCCTCAAG	59	152
	R: TTGCAGTAACTCGTCGGGTC		
PCNA	F: AATGCGGATACGTTGGCTCT	59	184
	R: CACCAATGTGGCTGAGGTCT		
CDK2	F: TCTTCCGTATCTTCCGCACG	59	190
	R: ATGCGCTTGTTGGGATCGTA		
Caspase 3	F: TTCCACCGAGATACCGGACT	60	179
	R: AAACTGCTTCGCTTGCTGTG		
Caspase 9	F: TCCCGGGCTGTTTCAACTT	60	207
	R: CCTCATCTTGCAGCTTGTGC		
Bax	F: GTGATGGCATGGGACATAGCTC	58	90
	R: TGGCGTAGACCTTGCGGATAA		
Col I	F: ATCAGGGCCGAAGGAAACAG	59	73
	R: ATGCTCCAGTGTGACTCGTG		
Col II	F: GCCACCCTCAAATCCCTCAA	57	246
	R: CACTCGGGATGGCAGAGTTT		
Sox 9	F: CGATTACACCGAGCACCAGA	58.5	338
	R: GTCTGACAGAAGTCCTCCACT		
ACAN	F: AACCTTCAGCATCTGGAGCC	59	154
	R: GAGGGAAGGCCACTTTCTCC		
Col X	F: GGCCAATCCACAATCCCAGA	59	148
	R: CCCCAGGGTAGGCTTTTGAG		
MMP13	F: GTTATGGACACAGGCTCCCC	59.5	165
	R: AGTATGCAGGATGCGGACAA		
gga-miR-205a	F: CGTCCTTCATTCCACCGG	59	
	R: AGTGCAGGGTCCGAGGTATT		
gga-miR-205a stem loop	GTCGTATCCAGTGCAGGGTCCGAGG TATTCGCACTGGATACGACCAGACT	50	
U6	F: TTCGGCAGCACATATACTAAAATTGGA	60	94
	R: CGAATTTGCGTGTCATCCTTGC		

### Western blotting assay

Following 48 h of transfection, total protein was extracted from chicken chondrocytes using RIPA reagent (Beyotime, Jiangsu, China), and the protein concentration was assessed using a BCA Protein Assay Kit (Beyotime). Subsequently, each protein sample was added with 1/4 ratio of the loading buffer (Coolaber, Beijing, China) and denatured at 100°C for 5 min. Then, the samples were separated by 10% sodium dodecyl sulfate-polyacrylamide gel electrophoresis (**SDS-PAGE**) and transferred to polyvinylidene fluoride (**PVDF**) membranes (Beyotime, Shanghai, China), which were blocked for 2 h at 4°C using a protein-free fast blocking solution (Servicebio, Wuhan, China). Col I (Abclonal, Wuhan, China; 1:1000 dilution), Col II (Abclonal, Wuhan, China; 1:500 dilution), Col X (Abclonal, Wuhan, China; 1:1000 dilution), and Tublin (Abclonal, Wuhan, China; 1:1000 dilution) were the primary antibodies, and corresponding secondary antibodies were goat anti-rabbit IgG (Zenbio, Chengdu, China; 511203; 1:5000 dilution) or goat anti-mouse IgG (Zenbio, Chengdu, China; 511103; 1:5000 dilution). Ultimately, ECL luminescent reagent (Beyotime, Shanghai, China) was used to carry out chemiluminescence imaging. Imag-Pro Plus 6.0 (Media Cybernetics, Maryland, USA) was used to assess the relative intensities of these bands. Three iterations of this experiment were conducted.

### CCK-8 assay and EdU assay

When the density of TD chondrocytes inoculated in 96-well plates reached 60-70%, miR-205a mimic, miR-205a inhibitor, pcDNA3.1-*RUNX2*, si-*RUNX2*, and their respective NC were transfected. After transfection, 10 μL of cell counting kit (**CCK-8**) reagent (RiboBio, Guangzhou, China) was added to each well at 12, 24, 36, and 48 h, followed by incubation in the incubator for an additional 2 h. Absorbance at various time points was measured at 450 nm using an enzyme-linked immunosorbent assay (ELISA) reader (Thermo Scientific, USA). Each experiment was conducted in triplicate.

To validate the results of CCK-8 assay, we employed the Cell-Light 5-ehtynyl-2’-deoxyuridine (**EdU**) Apollo In Vitro Kit (RiboBio, Guangzhou, China) to assess cell proliferation. In short, once the cell density in the 6-well plate reached 60-70%, miR-205a mimics, miR-205a inhibitor, pcDNA3.1-*RUNX2*, si-*RUNX2* and their corresponding NC were transfected. After 48 h transfection, 50 μM EdU reagent was added to each well and incubated for 2 h at 37°C. Subsequently, the cells were stained away from light with Hoechst 33,342 reaction solution for 25 min. In the same field of view, three random images were captured using an inverted fluorescent microscope (Nikon, Japan). Image-Pro Plus 6.0 software (Media Cybernetics, Inc., Rockville, MD, USA) was utilized to analyze the images, quantifying both the total number of cells and the number of EdU-positive cells.

### Cell cycle assay

When the density of chondrocytes inoculated in 12-well cell culture plates reached 60%-70%, transfection was performed. Following a 48 h transfection, the cells were collected and suspended in pre-cooled 75% ethanol overnight. Then, each cells sample was added with 500 μL of propidium iodide (**PI**, 50μg/mL, Beyotime, Shanghai, China) and incubated for 30 min at room temperature without light. BD FACSCanto II (BD Biosciences, USA) flow cytometry and FlowJo V10 software were used to examine the cell cycle. At least three replications of each experiment were conducted.

### Cell apoptosis assay

The Annexin V-FITC Apoptosis Detection Kit (Beyotime, Jiangsu, China) was utilized to assess the apoptosis of TD chondrocytes. Briefly, following a 48 h transfection period, the cells were inoculated in culture dishes, rinsed three times with pre-cooled PBS, resuspended in 100 μL binding buffer, and subsequently stained with the 5 μL Annexin V-FITC (Beyotime) and 5 μL PI (Beyotime) at 37°C for 10 min under dark conditions. Fluorescence intensity was measured using a BD FACSCanto II flow cytometer (BD Biosciences, Franklin Lakes, NJ, USA), and the data were statistically analyzed with FlowJo V10 software.

### Statistical analysis

SPSS statistical software (version 26.0, SPSS, Inc., USA) was utilized to analyze all data from this experiment, while GraphPad Prism software (version 8.0, GraphPad Software, Inc., USA) was employed to create experimental graphs. Differences between two groups were examined using one-way analysis of variance (**ANOVA**) or t-test. Data are presented as mean±standard error of the mean (**SEM**) from three independent experiments, with P-values less than 0.05 considered statistically significant.

## Results

### Identification and observation of chicken chondrocytes

**Fig. 1:**
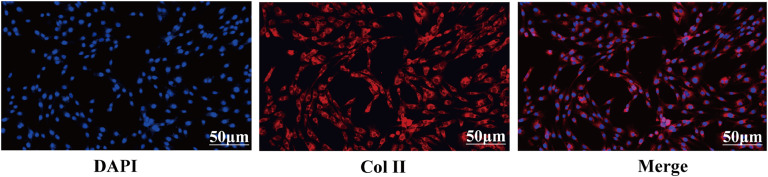
Chondrocyte immunofluorescence assay (200 ×). Nucleus stained with DAPI (blue). Chondrocyte markers *Col II* (red). Merged images are shown on the right.

To confirm that collected cells were chondrocytes, we performed immunofluorescence staining to assess the expression of collagen type II (***Col II***), a marker for mature chondrocytes. Fig. 1 clearly illustrates that DAPI-labeled nuclei exhibit bright blue fluorescence, while chondrocytes secrete *Col II*, which is prominently expressed in their cytoplasm. In summary, immunofluorescence staining validated that the primary cells we isolated were indeed chondrocytes.

### The effect of miR-205a on proliferation and apoptosis of TD chondrocytes

**Fig. 2:**
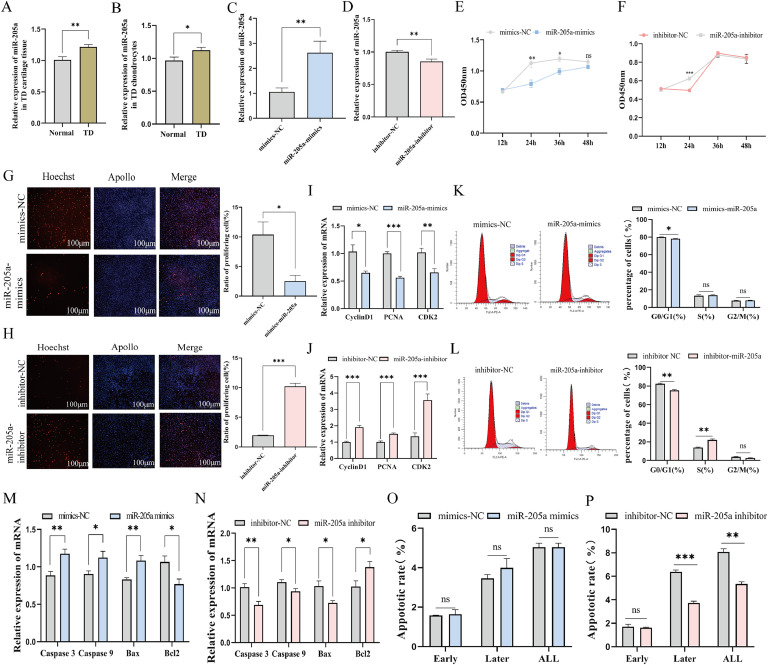
The effects of miR-205a on the proliferation and apoptosis of chondrocytes in TD broilers. (A, B) Expression levels of miR-205a in cartilage tissues and cells from both control group and TD groups. (C, D) miR-205a overexpression and interference efficiency. (E, F) The absorbance values of chondrocytes at 450 nm were measured at various time points following transfection with miR-205a mimics and inhibitor using the CCK-8 assay. (G, H) The EdU results of cell proliferation after transfection 48 h with miR-205a mimics and inhibitor. Scale bar=200 μm, magnification, *100. (I, J, M, N) The expression of genes related to proliferation and apoptosis following transfection with miR-205a mimics, inhibitor and their respective negative control. (K, L) The alterations in the cell cycle following transfection with miR-205a mimics and inhibitors were assessed using flow cytometry. (O, P) Flow cytometry results of Annexin V-positive apoptotic chondrocytes after transfection of miR-205a mimics and inhibitor, apoptosis scatter plots are shown in Fig S1. In all graphs, the results were indicated as mean**±**SEM, * *P*<0.05, **P<0.01, ***P<0.001 and ns means *P*≥0.05.

First, the qRT-PCR analysis demonstrated a significant increase in miR-205a expression in cartilage tissues of TD (*P*<0.05, Fig. 2A). To validate this observation, we cultured TD chondrocytes alongside normal chondrocytes and subsequently conducted real-time PCR analysis on these cells. The results indicated that miR-205a levels were notably higher in TD chondrocytes compared to the normal counterparts (*P*<0.01, Fig. 2B). Next, to elucidate the role of miR-205a in regulating proliferation and apoptosis in chicken TD chondrocytes, we transfected the cells with miR-205a mimics, miR-205a inhibitor, and their corresponding NC. Concurrently, the overexpression efficiency of miR-205a was significantly enhanced while its interference efficiency was notably reduced (*P*<0.01, Fig. 2C, D). CCK-8 assay was employed to assess the proliferation status of TD chondrocytes. We observed a significant decrease in optical density (**OD**) values at 24 and 36 h following transfection with miR-205a mimics, whereas OD value significantly increased at 24 h after transfection with miR-205 inhibitors (*P*<0.05, Fig. 2E, F). Similarly, miR-205a overexpression reduced EdU-positive cell numbers, while interference with miR-205a increased the number of EdU-positive cells (Fig. 2G, H). Additionally, cell cycle results shown that overexpression of miR-205a resulted in a reduction in the proportion of TD chondrocytes in the G0/G1 phase, accompanied by a slight increase in the S phase; however, this change difference is not significance (*P*>0.05, Fig. 2K). In contrast, interference with miR-205a elicited an opposing effect that was statistically significant (*P*<0.01, Fig. 2L). These findings were further corroborated by the expression of proliferation-related genes such as *CyclinD1, PCNA*, and *CDK2* (Fig. 2I, J). Moreover, overexpression of miR-205a resulted in an increased chondrocyte apoptosis rate (Fig. 2O, Fig.S1A), significantly upregulating the expression of pro-apoptotic genes *Caspase 3, Caspase 9* and *Bax*, while downregulating the expression of anti-apoptotic gene *Bcl_2_* (*P*<0.01, Fig. 2M). Conversely, interference with miR-205a decreased the apoptosis rate in chondrocytes (Fig. 2P, Fig. S1B), leading to a significant downregulation of *Caspase 3, Caspase 9* and *Bax* expressions, while the expression of *Bcl_2_* was significantly upregulated (*P*<0.01, Fig. 2N). These findings suggest that miR-205a inhibits proliferation while promoting apoptosis in TD chondrocytes.

### The effect of miR-205a on the differentiation of TD chondrocytes

**Fig. 3:**
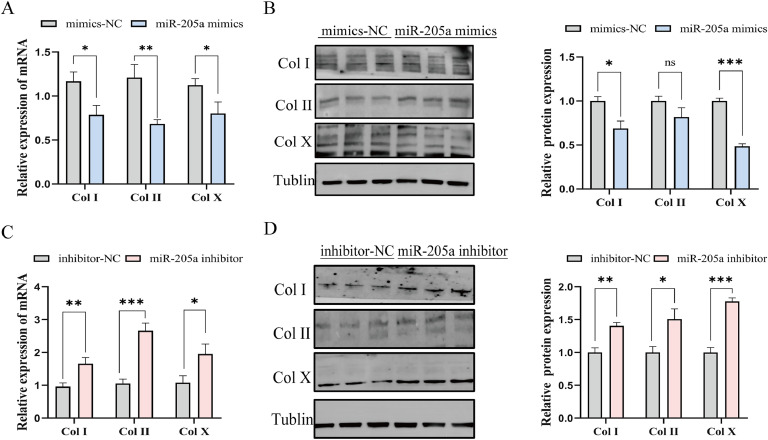
miR-205a inhibits TD chondrocytes differentiation. (A, C) The results of qRT-PCR for chondrocyte differentiation marker genes, including *Col I, Col II*, and *Col X*, following transfection with miR-205a mimics or miR-205a inhibitors over a 48-hour period (n=9). (B, D) The protein expression levels of Col I, Col II and Col X, as well as Tubulin proteins in TD chondrocytes following transfection with miR-205a mimics and inhibitors (n=3). The results are presented as mean±SEM, ns *P*≥0.05, *P<0.05, **P<0.01, ***P<0.001.

In present study, we examined the role of miR-205a in TD chondrocyte differentiation by transfecting cells with miR-205a mimics or inhibitors. The results demonstrated that the mRNA and protein expression levels of cartilage differentiation marker genes Col I and Col X were significantly reduced following overexpression of miR-205a (*P*<0.05, Fig. 3A, B), whereas interference with miR-205a produced an opposing effect (Fig. 3C, D). Therefore, these findings demonstrate that miR-205a inhibits differentiation in chicken TD chondrocyte.

### miR-205a directly target RUNX2

**Fig. 4:**
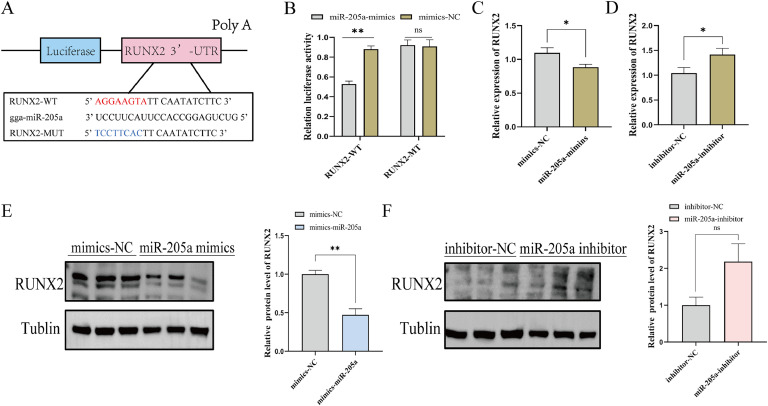
miR-205a directly targets RUNX2 in TD chondrocytes. (A) Prediction of the miR-205a binding site within the 3’UTR of *RUNX2*. (B) Dual-luciferase reporter assays were conducted following the co-transfection of miR-205a mimics with either wild-type (**WT**) or mutant vectors (n=3). (C, D) Relative mRNA expression levels of *RUNX2*4 following 48 hours of transfection with miR-205a mimics or inhibitors (n=9). (E, F) Western blotting analysis of RUNX2 protein expression following transfection with miR-205a mimics and inhibitor (n = 3). The results are shown as mean±SEM, ns *P*≥0.05, *P<0.05, **P<0.01.

To investigate the regulatory mechanism of miR-205a on TD chondrocytes in greater depth, we utilized RNAhybrid and miRDB online software to predicted the potential molecular targets of miR-205a, among which *RUNX2* was identified as a candidate target gene (Fig. 4A). Subsequently, we validated the interaction between the target genes using a dual-luciferase reporter assay. The results demonstrated that miR-205a mimics significantly reduced the luciferase activity of the wild-type RUNX2 plasmid; however, the dual fluorescence activity of the vector containing the mutated miR-205a binding site did not exhibit significantly changes (Fig. 4B). As anticipated, qRT-PCR and WB analyses demonstrated that when miR-205a was overexpressed, both the mRNA and protein levels of RUNX2 were significantly lower compared to the control group (*P*<0.05, *P*<0.01, Fig. 4C, E). However, interference with miR-205a resulted in increased expression of *RUNX2* (*P*<0.05, Fig. 4D, F). These findings collectively suggest that *RUNX2* is definitely a direct target gene of miR-205a in chicken TD chondrocytes.

### The effect of RUNX2 on proliferation and apoptosis of TD chondrocytes

**Fig. 5:**
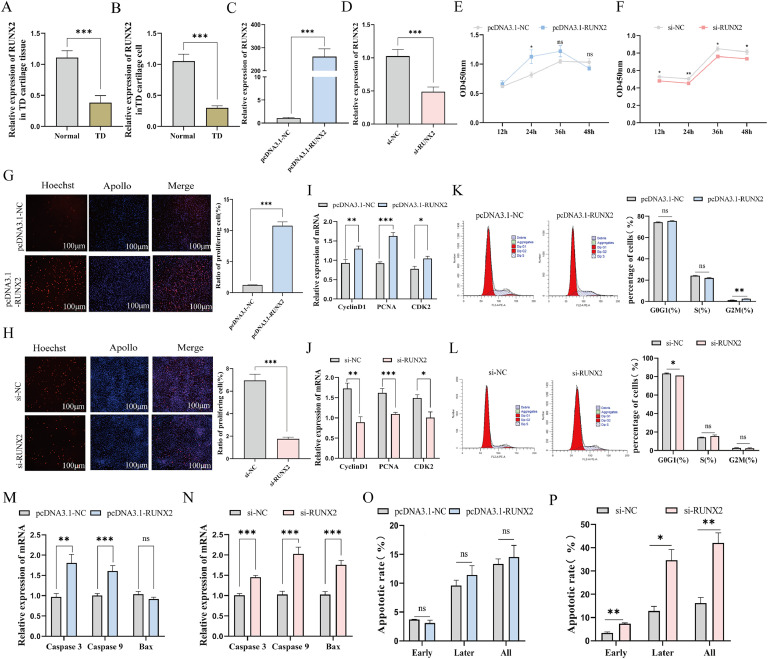
*RUNX2* promotes proliferation of TD chondrocytes. (A, B) Expression level of *RUNX2* was determined by qRT-PCR in cartilage tissues and cells of control and TD groups. (C, D) The expression level of *RUNX2* was evaluated by qRT-PCR in chicken chondrocytes that were transfected with either pcDNA3.1-*RUNX2* or si-*RUNX2*. (E, F) The cell proliferation of pcDNA3.1-*RUNX2* or si-*RUNX2* transfected for different time was observed by CCK-8 assay. (G, H) EdU assay was performed to detect the cell proliferation when transfected with pcDNA3.1-*RUNX2* or si-*RUNX2* for 48 h, along with positive cell number statistics. (scale bar: 100 μm, magnification, *100). (I, J) The relative expression levels of the proliferation-related genes in TD chondrocytes after transfection 48 h with pcDNA3.1-*RUNX2* or si-*RUNX2*. (M, N) Expression of mRNA of apoptosis-related genes after transfection with pcDNA3.1-*RUNX2* or si-*RUNX2*. (K, L) Percentage distribution of TD chondrocyte phases including G0/G1, S, and G2/M phases after transfection with pcDNA3.1-*RUNX2* or si-*RUNX2*. (O, P) Flow cytometry was performed to determine the early and late apoptotic rates of TD cells after transfection of pcDNA3.1-*RUNX2* or si-*RUNX2* in TD chondrocyte, apoptosis scatter plots are shown in Fig S2. Data were indicated as mean**±**SEM, ns *P*≥0.05, *P<0.05, ** *P*<0.01, ***P<0.001.

To explore how *RUNX2* plays a role in the development of TD in broilers, we evaluated the expression levels of *RUNX2* in tibial cartilage tissues and cells from both TD and control groups using qRT-PCR. The results showed a noticeable drop in *RUNX2* expression in both the cartilage tissues and cells from TD chickens (*P*<0.001, Fig. 5A, B). Subsequently, we transfected TD chondrocytes with pcDNA3.1-*RUNX2* and si-*RUNX2* (Fig. 5C, D). Subsequently, we conducted CCK-8 assays and EdU staining to assess cell proliferation status. The results indicated that overexpression of *RUNX2* enhanced both cell viability (Fig. 5E) and the count of EdU-positive cells (*P*<0.01, Fig. 5G), whereas silencing *RUNX2* produced opposing effects (Fig. 5F, H). Interestingly, after *RUNX2* was overexpressed, more TD chondrocytes moved into the G2/M phase (*P*<0.01, Fig. 5K), whereas interference with *RUNX2* resulted in an opposing outcome (*P*<0.05, Fig. 5L). Also, when *RUNX2* was overexpressed, it boosted the mRNA expression levels of genes related to cell proliferation, like *CyclinD1, PCNA*, and *CDK2* (*P*<0.05, Fig. 5I), whereas silencing *RUNX2* resulted in decreased expression levels of these genes (*P*<0.05, Fig. 5J). To further elucidate the biological effects of *RUNX2* on TD chondrocytes, we analyzed apoptosis via flow cytometry. Following overexpression of *RUNX2*, both late-stage apoptosis rates and total apoptosis rates among chondrocytes increased, but was not statistically significant (*P*>0.05, Fig. 5O, Fig. S2A). In contrast, interference with *RUNX2* significantly upregulated apoptosis rates across all three time points examined (*P*<0.05, Fig. 5P, Fig. S2B). As expected, overexpression *RUNX2* greatly increased *Caspase 3* and *Caspase 9* (*P*<0.01), and decreased *Bax* expression in TD chondrocytes (Fig. 5M), whereas the expression of *Caspase 3, Caspase 9*, and *Bax* increased significantly after *RUNX2* interference (*P*<0.01, Fig. 5N). These results indicate that *RUNX2* does not regulate TD chondrocyte apoptosis under normal conditions, but the rate of TD chondrocyte apoptosis increased when *RUNX2* was inhibited, which means that *RUNX2* inhibits TD chondrocyte apoptosis. These findings suggest that *RUNX2* promotes proliferation and inhibits apoptosis in TD chicken chondrocytes.

### Effect of RUNX2 on differentiation of TD chondrocyte

**Fig. 6:**
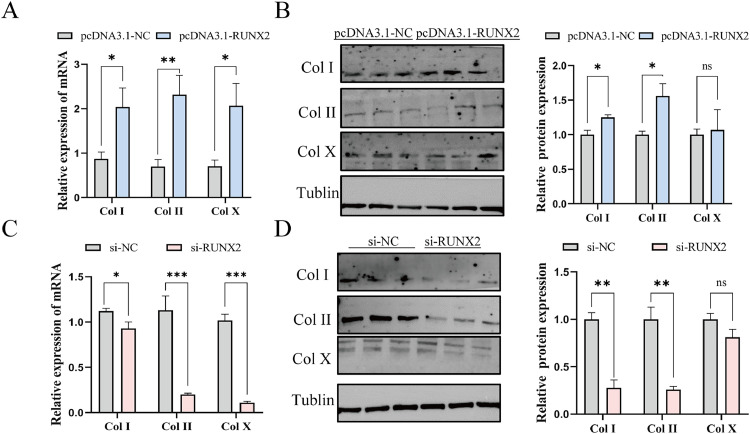
*RUNX2* promotes TD chondrocytes differentiation. (A, C) After 48 hours of transfection with pcDNA3.1-*RUNX2*, si-*RUNX2*, the expression levels of chondrodifferentiation-related genes in TD chondrocytes were evaluated (n=9). (B, D) The expression levels of chondrodifferentiation-related proteins in TD chondrocytes transfected with pcDNA3.1-*RUNX2* and si-*RUNX2* for 48h were assessed using WB analysis (n=3). The data were indicated as mean±SEM, ns *P*≥0.05, *P<0.05, ** *P*<0.01, ***P<0.001.

To explore how *RUNX2* affects chicken chondrocyte differentiation, we treated the cells with pcDNA3.1-*RUNX2*, si-*RUNX2*, and their matching negative controls. The result of the qRT-PCR analysis revealed that treatment with pcDNA3.1-*RUNX2* markedly enhanced the expression levels of *Col I, Col II*, and *Col X* in comparison to be the NC group (*P*<0.05, Fig. 6A). In contrast, the silencing of *RUNX2* using si-*RUNX2* led to a decrease in the expression levels of *Col I, Col II*, and *Col X* (*P*<0.05, Fig. 6C). Also, the WB analysis backed up these findings by showing similar trends in mRNA expression levels (Fig. 6B, D). Overall, these data suggest that *RUNX2* is important for helping chicken TD chondrocytes to differentiate.

### miR-205a inhibits cell proliferation and differentiation via RUNX2

**Fig. 7:**
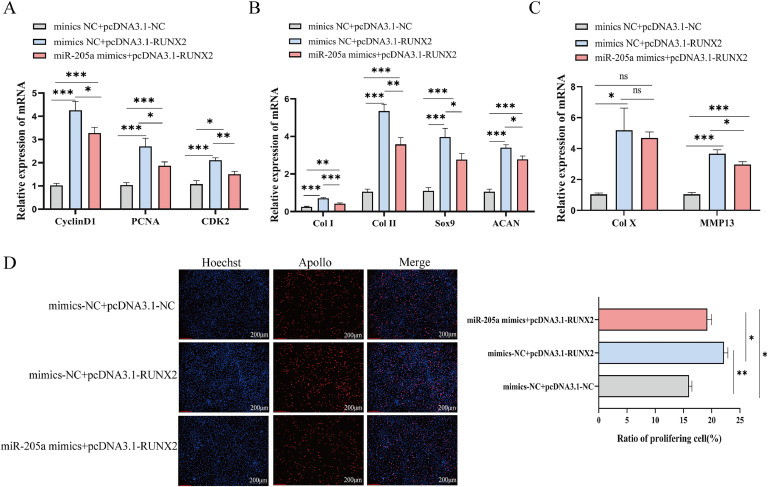
miR-205a inhibits chondrocyte proliferation and differentiation by down-regulating the expression of *RUNX2*. (A) The mRNA expression of the same proliferation-related genes after (co-) overexpression of miR-205a and/or *RUNX2*, as specified in the legend. (B, C) The mRNA expression of differentiation-related genes in TD chondrocytes was assessed by qRT-PCR after (co-) overexpression of miR-205a and/or *RUNX2*. (D) After (co-) overexpression of miR-205a and/or *RUNX2,* EdU analyzed the proliferation of TD chondrocytes, along with positive cell number statistics (scale bar: 200 μm). The data were indicated as mean±SEM (n=3), ns *P*≥0.05, *P<0.05, ** *P*<0.01, ***P<0.001.

To further validate the role of *RUNX2*, we conducted in vitro rescue experiments. The results from qRT-PCR indicated that, compared to the group co-transfected with mimics NC and pcDNA3.1-*RUNX2*, the expression levels of proliferation-related genes *CyclinD1, PCNA*, and *CDK2* were significantly reduced in TD chondrocytes co-transfected with miR-205a mimics and pcDNA3.1-*RUNX2* (*P*<0.05, Fig. 7A). Additionally, EdU assays corroborated these findings (Fig. 7D). Moreover, co-transfection of miR-205a mimics along with pcDNA3.1-*RUNX2* led to a decrease in the expression levels of differentiation-related genes such as *Col I, Col II, Col X, SOX9, ACAN*, and *MMP13* when compared to the group co-transfected with mimics NC and pcDNA3.1-*RUNX2* (Fig. 7B, C). These results indicate that miR-205a slows down chondrocyte growth and differentiation by lowering *RUNX2* expression.

## Discussion

TD is one of the most common leg diseases in broilers and is known to be a multifactorial disease affected by factors like breed, feeding management, nutrition and genetics (Praul et al., 2000; Lu et al., 2022). The occurrence of TD not only compromises broiler production performance but also negatively impacts meat yield, quality, and reproductive efficiency in chickens (Huang et al., 2021), thereby exacerbating the challenges in broiler breeding. In our prior transcriptomic analysis of cartilage tissues from both TD-affected and healthy chickens, we elucidated the involvement of circRNAs, miRANs, and mRNAs in critical biological processes such as cartilage development, cell proliferation and differentiation (Lu et al., 2022). miRNA have been shown to regulate a diverse array of cellular processes, including growth, proliferation, differentiation, apoptosis, and metabolism (Tang et al., 2008b). Recent research has revealed that several miRNAs involved in osteoblast differentiation, such as miR-33-5p and miRNA-194 (Wang et al., 2016; Li et al., 2015). Importantly, miRNA dysfunction has been associated with various bone-related diseases (Taipaleenmäki, 2018). Therefore, investigating the biological functions of aberrantly regulated miRNAs in chicken TD may facilitate the identification of novel biomarkers and therapeutic strategies for bone diseases in poultry. In our previous study, we observed that miR-205a was differentially expressed in cartilage tissues and cells of control and TD groups (Lu et al., 2022). This prompted us to conduct a more in-depth investigation into the role of miR-205a in regulating the growth and differentiation of TD chondrocytes from a miRNA perspective. Building on these insights, we investigated the biological functions of miR-205a in TD chondrocytes to establish a theoretical framework for elucidating the molecular mechanisms underlying the pathogenesis of TD. miRNA-205 is highly conserved across various germlines (Plantamura et al., 2020). Numerous studies have established a link between the expression of miRNA-205 and the development of multiple tumors (Qin et al., 2013), although its biological role varies among different tumor types. It has been demonstrated that miR-205 is downregulated and exerts tumor suppressor effects in various cancer types. For instance, in bone metastatic prostate cancer, levels of miRNA-205 were further reduced compared to those in non-bone metastatic prostate cancer (Sun et al., 2020). Conversely, in non-small cell lung cancer, levels of miR-205-5p were significantly elevated and acted as an oncogene by downregulating erbB3 expression (Jiang et al., 2017). In this study, we identified a significant elevated in the expression of miR-205a in TD cartilage tissue and chondrocytes compared to their normal counterparts. Furthermore, overexpression of miR-205a inhibited proliferation and differentiation, but promoted apoptosis in TD chondrocytes, as evidenced by flow cytometry results along with the upregulation of *Caspase 3, Caspase 9*, and *Bax*, coupled with downregulation of *Bcl_2_*. Apoptosis can be triggered through two distinct pathways: the extrinsic pathway and the intrinsic pathway (Fulda, 2018). The intrinsic pathway is tightly regulated by members of the *Bcl_2_* family (Kale et al., 2018). Therefore, it is plausible that miR-205a may induce apoptosis in TD chondrocytes via the intrinsic pathway.

We utilized bioinformatics tools to identify potential target genes of miR-205a and elucidate the mechanisms through which miR-205a regulates TD. Among the potential targets, we decided to look at *RUNX2* because of its known role in the growth and development of chondrocytes and osteoblasts (Komori, 2022). *RUNX2* is essential for the differentiation, maturation, and proper functioning of osteoblasts, chondrocytes, and mesenchymal stem cells (Ziros et al., 2002; Komori, 2020; Gargalionis et al., 2024). In this study, we validated *RUNX2* as a target of miR-205a through the application of a luciferase reporter assay. We observed that overexpression of miR-205a inhibited RUNX2 mRNA and protein expression in TD chondrocytes. Furthermore, our analysis revealed a negative correlation between *RUNX2* expression levels and those of miR-205a in TD chondrocytes, indicating that *RUNX2* is indeed a direct target gene of miR-205a within this context. Moreover, we explored the influence of *RUNX2* on TD chondrocytes and uncovered that it significantly enhances their proliferation and differentiation. This finding is consistent with prior studies that illustrate how *RUNX2* overexpression expedites chondrocyte maturation and differentiation across the cartilage (Komori, 2020). Similarly, mice deficient in *RUNX2* exhibit an absence of osteoblasts and impaired bone formation alongside significantly reduced chondrocyte maturation and development (Kim et al., 1999; Komori, 2019). In light of these findings, we contend that miR-205a plays a significant role in the initiation and progression of TD through its targeted regulation of *RUNX2*.

Nevertheless, an intriguing phenomenon arose from our study: when RUNX2 was overexpressed, it not only boosted the growth and development of chondrocytes but also enhanced the expression of specific apoptotic genes. This observation necessitates a re-evaluation of the process of bone formation in chondrocytes. Endochondral ossification is the principal process underlying tibial development and growth in chickens, encompassing chondrocyte proliferation, differentiation, hypertrophy, and subsequent apoptosis (Kronenberg H M, 2003). During this process, chondrocytes transition from the proliferation phase to the differentiation stage, ultimately maturing into hypertrophic chondrocytes. These hypertrophic chondrocytes undergo regulated apoptosis, thereby creating spaces that facilitate the invasion of blood vessels and osteoblasts. Subsequently, the apoptotic chondrocytes are resorbed, and the vacated spaces are occupied by osteoblasts, resulting in the formation of new bone tissue (Kronenberg H M, 2003). However, if chondrocytes do not undergo proper apoptosis, there may be an abnormal accumulation of hypertrophic chondrocytes, which can lead to dysregulated apoptosis or even cell death (Farquharson and Jefferies, 2000). It is crucial to underscore that this study demonstrates the adverse effects of miR-205a overexpression and *RUNX2* inhibition on the treatment and recovery of TD. Our findings reveal that both the overexpression of miR-205a and the silencing of *RUNX2* significantly slow down the growth and development chondrocytes. Cell cycle analysis showed a noticeable drop in the number of cells in the G0/G1 phase, which suggests that even though chondrocytes are activated, they are stuck in this phase. This arrest may account for the observed upregulation of apoptosis-related genes, this phenomenon was also reported by Cao et al. (2018). In contrast, the overexpression of *RUNX2* markedly enhanced chondrocyte proliferation and differentiation capacity, as well as the proportion of cells in the G2/M phase. Although there was an observed increase in both late apoptosis and total apoptosis rates though they were not statistically significant. These findings indicated that *RUNX2* overexpression facilitates the advancement of chondrocyte development, potentially contributing to the amelioration of TD. These insights imply that inhibition of miR-205a or overexpression of *RUNX2* could enhance chondrocyte development potentially contributing positively towards ameliorating TD conditions. However, further validation by in vivo studies may be needed in the future to elucidate the precise regulatory roles and specific mechanisms by which miR-205a and *RUNX2* affect TD pathogenesis and progression.

## Conclusion

In conclusion, our findings suggest that miR-205a expression is upregulated in TD chondrocytes and tissues, while RUNX2 expression is downregulated. Furthermore, miR-205a plays a crucial role in inhibiting proliferation and differentiation through its target gene *RUNX2* in chicken TD chondrocytes. This study underscores the significance of miR-205a in the pathogenesis of TD for the first time, offering novel insights into the regulatory mechanisms underlying miR-205a-mediated TD development. Additionally, it presents a promising target and strategy for breeding varieties resistant to TD.

## Declaration of competing interest

The authors declare that they have no known competing financial interests or personal relationships that could have appeared to influence the work reported in this paper.

## Appendix Supplementary materials

Image, application 1
