# Supplementary material for: The effect of miR-205a with RUNX2 towards proliferation and differentiation of chicken chondrocytes in thiram-induced tibial dyschondroplasia
Source: Poult Sci. 2024 Nov 8;103(12):104535. doi: 10.1016/j.psj.2024.104535 (PMC11609359; doi:10.1016/j.psj.2024.104535)
Supplement: Supplementary file 1 [file mmc1.docx]

**Supporting information**

**The effect of miR-205a with RUNX2 towards proliferation and differentiation of chicken chondrocytes in thiram-induced tibial dyschondroplasia**

Yuxin Zhou^1,2,3†^, Yuxiang Lu^1,2,3†^, Hengyong Xu^1,2,3†^, Xuyang Ji^1,2,3^, Qingqing Deng^1,2,3^, Xi Wang^1,2,3^, Yao Zhang^1,2,3^, Qiuhang Li^1,2,3^, Yusheng Lu^1,2,3^, Alma Rustempasic^4^, Yiping Liu^1,2,3^, Yan Wang^1,2,3*^

*^1^State Key Laboratory of Swine and Poultry Breeding Industry, College of Animal Science and Technology, Sichuan Agricultural University, P. R. China, Chengdu 611130;*

*^2^Farm Animal Genetic Resources Exploration and Innovation Key Laboratory of Sichuan Province, Sichuan Agricultural University, Chengdu Campus, Chengdu 611130, China;*

*^3^Key Laboratory of Livestock and Poultry Multi-omics, Ministry of Agriculture and Rural Affairs, College of Animal Science and Technology, Sichuan Agricultural University, P. R. China, Chengdu, 611130.*

*^4^Faculty of Agriculture and Food Science, University in Sarajevo, Zmaja od Bosne 8, 71000 Sarajevo, Bosnia and Herzegovina*

^*^To whom correspondence should be addressed. Tel: +86-028-86291010; Fax: +86-028-86291010, E-mail: [as519723614@163.com](mailto:as519723614@163.com)

^†^These authors contributed equally to this work.

**
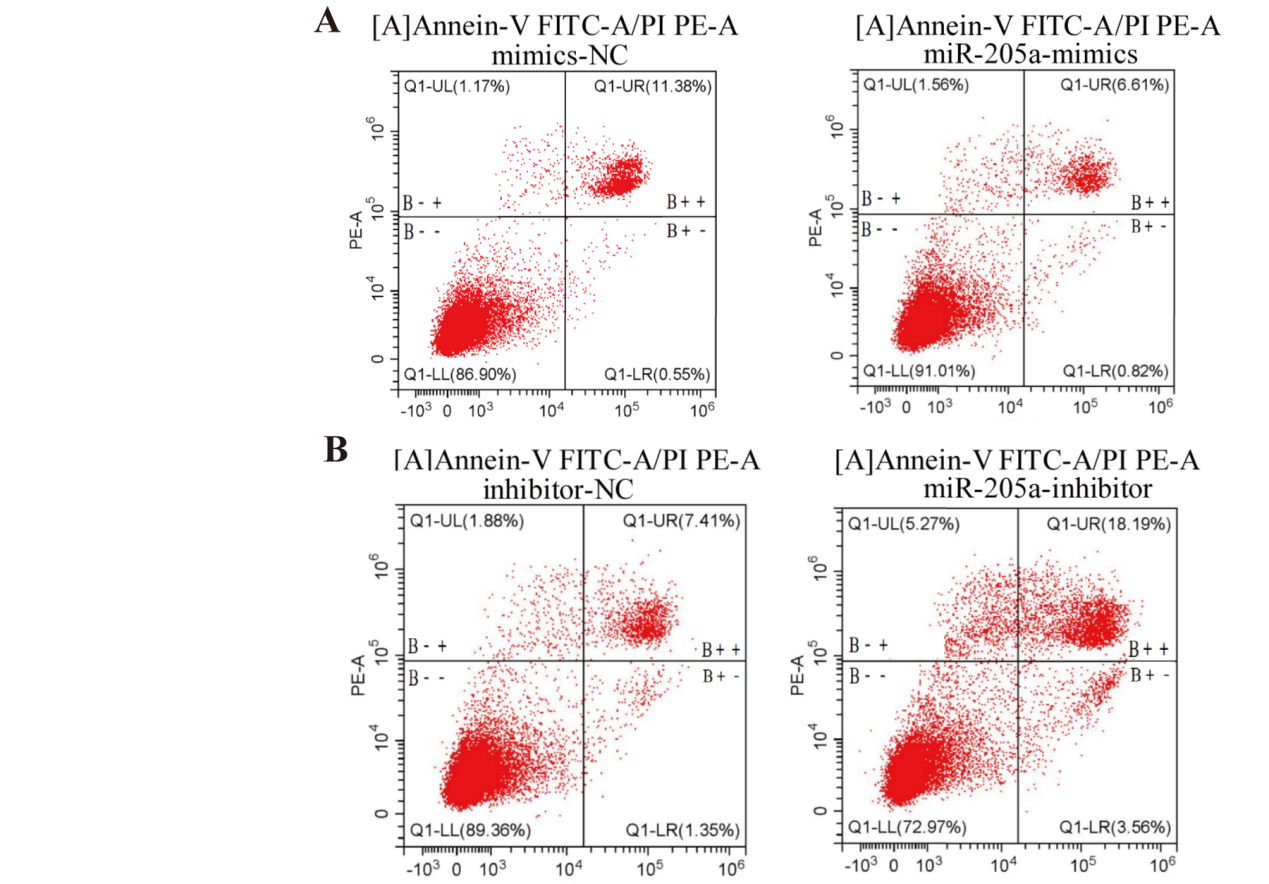
**

**Fig. S1.** Scatter plot of apoptosis rate of TD cells after transfection with miR-205a mimics and miR-205a inhibitor. (A) Transfection with mimic NC and miR-205a mimics; (B) Transfection with inhibitor NC and miR-205a inhibitor.


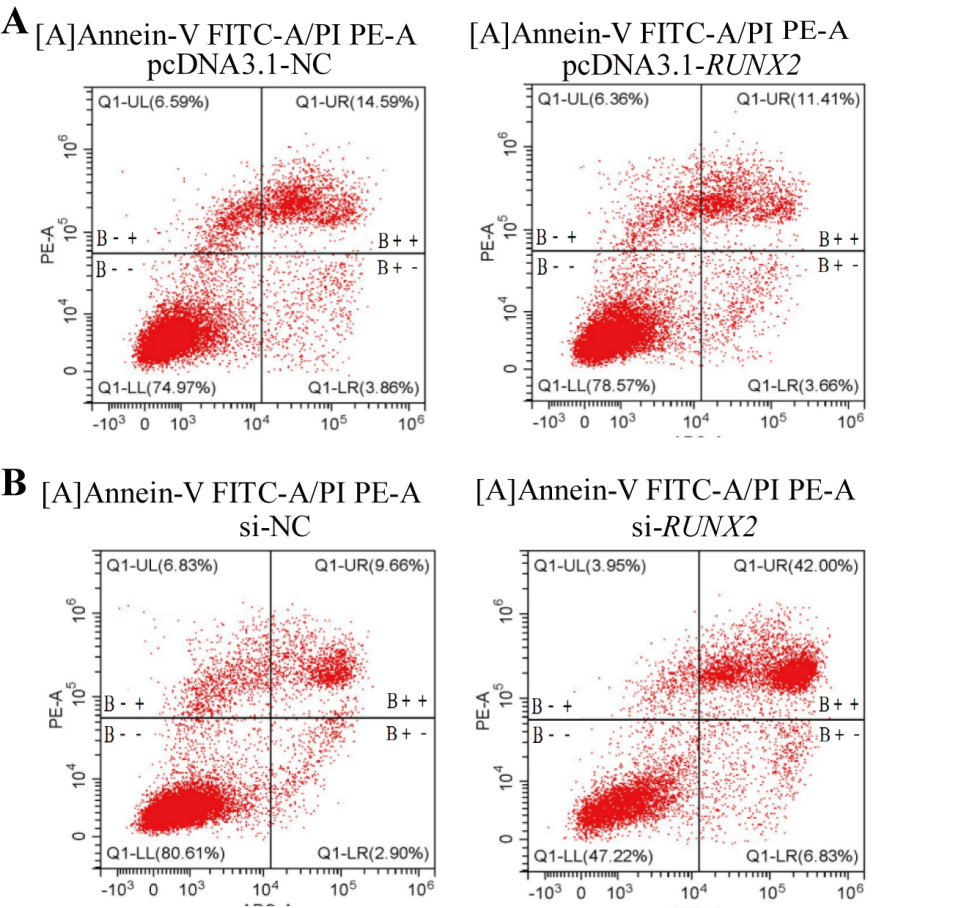


**Fig. S2.** Scatter plot of apoptosis rate of TD cells after transfection with pcDNA3.1-*RUNX2* and si-*RUNX2*. (A) Transfection with pcDNA.1-NC and pcDNA3.1-*RUNX2*; (B) Transfection with si-NC and si-*RUNX2*.
